# Long-term cellular and humoral responses to SARS-CoV-2 vaccinations in patients with solid malignancies undergoing chemotherapy

**DOI:** 10.3389/fimmu.2025.1690495

**Published:** 2026-01-29

**Authors:** Andrew Liu, Brian Necela, Zhuo Li, Davitte Cogen, Mikolaj A. Wieczorek, Ashita Mummareddy, Marites Acampora, Gina A. Reynolds, Pooja P. Advani, Alvaro Moreno-Aspitia, Melanie D. Swift, Abinash Virk, Adil E. Bharucha, Christopher P. Marquez, Tushar C. Patel, Keith L. Knutson, Saranya Chumsri

**Affiliations:** 1 Division of Hematology and Medical Oncology, Mayo Clinic, Jacksonville, FL, United States; 2 Department of Cancer Biology, Mayo Clinic, Jacksonville, FL, United States; 3 Division of Quantitative Health Science, Mayo Clinic, Jacksonville, FL, United States; 4 Department of Preventive, Occupational and Aerospace Medicine, Mayo Clinic, Jacksonville, FL, United States; 5 Division of Infectious Disease, Mayo Clinic, Jacksonville, FL, United States; 6 Division of Gastroenterology & Hepatology, Mayo Clinic, Jacksonville, FL, United States; 7 Department of Laboratory Medicine and Pathology, Mayo Clinic, Jacksonville, FL, United States; 8 Department of Immunology, Mayo Clinic, Jacksonville, FL, United States

**Keywords:** chemotherapy, humoral and cellular responses to routine vaccines, magnitude and durability, patients with solid tumors, SARS-CoV-2 vaccines

## Abstract

**Background:**

While SARS-CoV-2 vaccines are highly effective in healthy individuals, the magnitude and durability of humoral and cellular responses in patients with solid malignancies receiving chemotherapy remain understudied. Previous reports suggest that patients with cancer may not mount adequate immune response after SARS-CoV-2 vaccination. Additionally, most studies focused on humoral responses, while data regarding cellular immune responses are scarce. In this study, we evaluated humoral and cellular responses in patients with solid tumors receiving chemotherapy compared to healthy individuals up to one year after vaccinations.

**Methods:**

Patients aged ≥18 who were willing to receive the SARS-CoV-2 vaccine were enrolled. Anti-SARS-CoV-2 immunoassays were used to detect antibodies against nucleocapsid and spike proteins. Human IFN-γ Fluorospot assay was used to determine antigen−specific T-cell responses. Data were compared between groups using Mann−Whitney test for continuous variables and Fisher’s exact test for categorical variables.

**Results:**

A total of 67 subjects (47 patients with cancer and 20 healthy individuals) were included. 1–3 months following the second vaccine doses, 96% of patients with cancer and 100% of healthy individuals demonstrated a positive humoral response. While the positivity rate was not significantly different, patients with cancer had significantly lower spike IgG antibodies than healthy individuals. However, this difference diminished at 6 months when patients with cancer had increased antibodies compared to decreased antibodies in the healthy cohort and no difference was noticed at 12-month. Patients with cancer developed a similar antigen-specific T-cell response as healthy individuals at 1–3 months, 6 months and 12 months. There were no significant differences when comparing patients aged ≤ 55 years vs. >55 years, stages I-III vs. IV, single vs. multiple chemotherapy, and BNT162b2 vs. mRNA-1273 vaccines. There was a significant moderate correlation between neutralization and antibody levels at 12 months. However, despite patients with cancer having a significantly higher COVID risk score, there were no significant differences in COVID-19 infection and hospitalization between patients with cancer and healthy individuals.

**Conclusion:**

Despite initial impaired antibody responses to SARS-CoV-2 vaccinations, patients with solid malignancies receiving chemotherapy effectively generated long-term cellular and humoral responses by 6 and 12 months, leading to similar infection and hospitalization rates compared to healthy individuals.

## Introduction

The advent of SARS-CoV-2 vaccines has played a critical role in transforming the outcomes of the COVID-19 pandemic. Several studies demonstrated the remarkable efficacy of SARS-CoV-2 vaccines with robust and durable immune responses in healthy individuals (1–5). There are two major types of adaptive immune responses to vaccines, namely humoral and cellular immunity. Humoral immunity involves viral-specific antibodies, which prevent viral entry and acquisition of infection. Cellular immunity involves viral-specific T- and B-cell responses and provides long-term immunologic protection against infection (6–8). While the humoral immune responses through neutralizing antibodies are imperative in preventing infection by blocking viral entry, the cellular responses are critical in controlling infections and preventing severe disease and hospitalization (7, 9). Previous studies demonstrated the durability of neutralizing antibodies and antigen-specific memory B and T cells that persisted for at least 4–6 months following SARS-CoV-2 vaccinations in healthy individuals, particularly after receiving additional booster vaccinations (10–13).

Several studies demonstrated that patients with cancer have poorer outcomes with COVID-19 infection compared to healthy individuals (14, 15). Prior to the availability of SARS-CoV-2 vaccines, patients with cancer were reported to have alarmingly high mortality rates of up to 25-40%, especially among patients who were hospitalized (16–19). Furthermore, patients with cancer were reported to have a high incidence of long-term COVID-19 sequelae of 15-30% (20). While SARS-CoV-2 vaccines are highly effective in healthy individuals, their effectiveness in immunocompromised patients remains less known. Emerging data suggest that patients with cancer may not mount adequate protective immune response after SARS-CoV-2 infection (21) and after vaccination with SARS-CoV-2 vaccines (22–28). Most of the published studies focused on measuring humoral responses by assessing SARS-CoV-2 spike antibodies and the neutralizing capacity of these antibodies against emerging variants of concern (VoCs). As outlined in a previous review, data regarding T-cell response after SARS-CoV-2 vaccination in patients with cancer remains scarce (20). Additionally, most studies involved patients with cancer undergoing various types of therapy, which may have affected their immune systems differently, leading to different outcomes (20, 29, 30). Furthermore, longitudinal immune response data with longer follow-ups of more than 6 months in patients with cancer is also limited.

In this study, we evaluated both humoral and cellular immune responses in patients with solid malignancy receiving cytotoxic chemotherapy compared to healthy individuals up to 1 year after completion of the initial vaccinations. In addition to evaluating the level of SARS-CoV-2 spike antibody, we also quantitatively evaluated T cells responses using the IFN-γ fluorospot to measure peripheral antigen-specific T cell frequencies after stimulation with pools of SARS-CoV-2 spike peptides from the original strain as well as emerging Omicron variant (BA.5) in patients with solid malignancy receiving cytotoxic chemotherapy.

## Methods

### Patient selection

Patients ≥ 18 years old who were willing to receive COVID-19 vaccination from Mayo Clinic Florida were enrolled in this study between April and November 2021 However, due to rapid uptake of COVID-19 vaccination and slow accrual, the protocol was subsequently amended to allow patients who previously received 1 or 2 doses of COVID-19 vaccination within 3 months. For the cancer cohort, patients with histologically confirmed solid malignancy who were on or would be starting systemic cytotoxic chemotherapy were enrolled. For the healthy cohort, patients must not have a history of active malignancy ≤ 3 years, except for adequately treated non-melanotic skin cancer or carcinoma *in situ* of the cervix. However, if there was a history of prior solid tumor malignancy, it must have been treated curatively with no evidence of recurrence ≥5 years. Immunocompromised patients, including patients known to be HIV positive or those on immunosuppressive therapy other than chemotherapy, were excluded.

This study was approved by the Mayo Clinic Institutional Review Board and was conducted in compliance with the Good Clinical Practice, Declaration of Helsinki, and the International Conference on Harmonization. Written informed consent was voluntarily obtained from all participants.

### Study procedures

Patients who were enrolled had up to 5 blood draws for both humoral and cellular responses at baseline prior to the first vaccination, prior to the second vaccination (7–day window), 1–3 months (± 14 days), 6 months (± 30 days), and 12 months (± 30 days) after the last vaccination. However, due to the rapid uptake of SARS-CoV-2 vaccination, the protocol was amended in August 2021 to allow patients who already received 1 or 2 doses of SARS-CoV-2 vaccination prior to enrollment if it has not been longer than 3 months since the completion of the vaccination. Peripheral blood mononuclear cells (PBMCs) were isolated from heparinized whole blood using SepMate-50 isolation tubes (STEMCELL) per manufacturer instructions and cryopreserved in liquid nitrogen in RPMI-1640 containing 12.5% Serum Albumin Human, Fraction IV, 1.25% HEPES and 10% DMSO. The serum was prepared from whole blood by standard clotting procedure and immediately frozen at −80°C.

### Humoral immune responses

SARS-CoV-2 nucleocapsid and spike protein-specific IgG antibodies were measured in sera at baseline and at 1-, 6-, and 12-month post-vaccine timepoints in all patients using Elecsys^®^ Anti-SARS-CoV-2 and Anti-SARS-CoV-2 S (Roche Diagnostics) immunoassays that detect antibodies against nucleocapsid and spike proteins, respectively. Patients with positive COVID-19 infection, either prior or concurrent, were excluded from the study, as assessed with the nucleocapsid immunoassay.

### Neutralizing antibody responses

Neutralizing antibody activity of serum from patients 12 months post-vaccination was measured in a well-documented assay that measures the ability of antibodies to block/neutralize the entry of SARS-CoV-2 pseudoviral lentivirus containing a firefly luciferase (Luc) reporter gene into 293-ACE2 stable cell (31). Quantitative measurements of infection/neutralization were determined by measurement of luciferase. The assay was performed by incubating a 1:400 dilution of the patient’s serum with a 1:10 dilution of SARS-CoV-2 Omicron Ba.5 variant pseudovirus stock (ProSci, Poway CA) in a total volume of 100 µl of complete media (DMEM + 10% FBS), all performed in triplicate. The mix was incubated for 30 min at 37°C before being added to human HEK293/ACE2 cells (ACROBiosystems) freshly plated at 4E4 cells/well (100 µl total) in a white microplate. After 48 hours, luminescence values (RLU) were measured using the Britelite plus Reporter Gene Assay System (Perkin Elmer) and Turner Biosystems (Promega) luminometer. Neutralization was calculated using the formula $\%\;Neutralization=\left(1-\frac{\bar{CC}}{\bar{VC}}\right)\times$ 100, where $\bar{cc}$ = mean value of test group (pseudovirus + serum + cells) and $\bar{vc}$ = mean value of virus control group (pseudovirus + cells).

### T-cell response assay

Human IFN- γ FluorospotPlus assays (Mabtech) were performed to quantitate antigen-specific T cells capable of secreting interferon (IFN)-γ in response to a peptide pool covering the sequence of the spike protein of SARS−CoV−2. Cryopreserved peripheral blood mononuclear cells (PBMCs) were recovered by rapid thawing in a 37°C water bath and washed twice with pre-warmed (37°C) complete RPMI media containing 10% AB serum (Gemini Bio-Products) through centrifugation at 200g for 10 mins. PMBC samples with >85% viability were plated at 2.5 × 10^5^ per well in pre-blocked Fluorospot plates and incubated for 24 hours with complete medium alone, spike peptide pool (1ug/ml PepTivator^®^ SARS-CoV-2 Prot_S, Miltenyi Biotech), or phytohemagglutinin (PHA) (10 µg/mL, positive control). After washing with 1X PBS, plates were incubated with biotinylated anti-IFN-γ followed by streptavidin-550 conjugates, with washes between each step according to the manufacturer’s protocol. After the final wash, plates were incubated for 15 minutes with fluorescence enhancer-II and dried before reading on the Mabtech Iris™ reader. Antigen-specific T cells were defined as the average number of spots elicited by the spike peptide pool minus the average number of spots elicited with the control culture medium alone. All spot numbers were multiplied by four to achieve standardized spots per million cells. PHA was used as a positive control to ensure high T-cell responsiveness.

### Statistical analysis

SARS-CoV-2 spike antibody level, cellular T cell response, and % antibody neutralization were reported as median (range) and mean (standard deviation). Data were compared between groups with and without specific risk factors using the Wilcoxon rank sum test for continuous variables and Fisher’s exact test for categorical variables. Data were compared between groups using the Mann−Whitney test for continuous variables and Fisher’s exact test for categorical variables. All tests were two-sided, with a p-value of< 0.05 considered statistically significant. The analysis was done using R4.0.3.

## Results

### Patient characteristics

A total of 74 patients were enrolled, including 53 patients with solid malignancy who received cytotoxic chemotherapy and 20 healthy individuals between April and November 2021 at Mayo Clinic Cancer Center in Florida, USA. One patient was a screen fail, and 6 patients with cancer were excluded due to previous or concurrent COVID-19 infection measured by nucleocapsid immunoassay. 47 patients with cancer were included in the final data analysis. Patient characteristics in both cohorts, with 47 eligible patients with cancer and 20 healthy controls, are shown in **Table 1**. The median age of patients with cancer was 55.4 (range 29.9-81.4) years, with 8 (17%) male and 39 (83%) female. The type of chemotherapy was shown in supplemental **Table 1**. The median duration of chemotherapy was 5.1 months. For the healthy group, the median age was 49.6 (range 19.9-60.4) years, with 9 (45%) male and 11 (55%) female. The cancer cohort was significantly older (*P* = 0.027), with more female patients (*P* = 0.029). There was no statistically significant difference in body mass index as well as other comorbidities, including diabetes, hypertension, chronic lung condition, cardiac disease, obesity and liver disease. As expected, patients in the cancer cohort had significantly higher COVID-19 risk scores than healthy individuals (median = 2 vs 1, *P* = 0.003). The majority of patients (n = 51, 76.1%) received BNT162b2 (Pfizer), followed by 15 patients (22.4%) having received mRNA-1273 (Moderna), and 1 cancer patient having received Ad26.COV2.S (Johnson & Johnson). In the cancer cohort, the majority of patients had breast cancer (n = 31, 66%), followed by GI cancer (n = 11, 23.4%), lung cancer (n = 1, 2.1%), and others. For stage, 24 patients (51.1%) had stage IV or metastatic disease, and 23 (48.9%) had stage I-III. Most patients had a combination of cytotoxic chemotherapy (n = 30, 63.8%), 17 patients (36.2%) had single-agent chemotherapy, and 8 patients (17%) had chemotherapy in combination with an immune checkpoint inhibitor. The majority of patients had Eastern Cooperative Oncology Group performance status (ECOG PS) 0 in 31 patients (66%), ECOG PS 1 in 15 patients (31.9%), and ECOG PS 2 in 1 patient (2.1%).

**Table 1:** Baseline characteristics by cohort.

Baseline characteristics	Patients with cancer (N = 47)	Healthy control (N = 20)	Total (N = 67)	p value
Age at 1st vaccine (in years)				0.027
- N	47	20	67	
- Median (Range)	55.4 (29.9, 81.4)	49.6 (19.9, 60.4)	54.2 (19.9, 81.4)	
- Mean (SD)	55.4 (12.0)	47.2 (12.4)	52.9 (12.6)	
Age at 1st vaccine (>55)				0.437
-<=55	23 (48.9%)	12 (60.0%)	35 (52.2%)	
- >55	24 (51.1%)	8 (40.0%)	32 (47.8%)	
Sex				0.029
- Male	8 (17.0%)	9 (45.0%)	17 (25.4%)	
- Female	39 (83.0%)	11 (55.0%)	50 (74.6%)	
Race				0.005
- White	40 (85.1%)	10 (50.0%)	50 (74.6%)	
- Other	7 (14.9%)	10 (50.0%)	17 (25.4%)	
Body Mass Index				0.612
- N	47	20	67	
- Median (Range)	25.8 (17.6, 41.8)	26.0 (17.8, 42.1)	25.8 (17.6, 42.1)	
- Mean (SD)	26.4 (5.7)	27.6 (6.3)	26.8 (5.9)	
Diabetes				1.000
- Yes	6 (12.8%)	3 (15.0%)	9 (13.4%)	
- No	41 (87.2%)	17 (85.0%)	58 (86.6%)	
Hypertension				0.760
- Yes	12 (25.5%)	4 (20.0%)	16 (23.9%)	
- No	35 (74.5%)	16 (80.0%)	51 (76.1%)	
Chronic lung condition				1.000
- Yes	3 (6.4%)	1 (5.0%)	4 (6.0%)	
- No	44 (93.6%)	19 (95.0%)	63 (94.0%)	
Cardiac disease				1.000
- Yes	2 (4.3%)	1 (5.0%)	3 (4.5%)	
- No	45 (95.7%)	19 (95.0%)	64 (95.5%)	
Obesity				1.000
- Yes	13 (27.7%)	6 (30.0%)	19 (28.4%)	
- No	34 (72.3%)	14 (70.0%)	48 (71.6%)	
Liver disease				1.000
- Yes	1 (2.1%)	0 (0.0%)	1 (1.5%)	
- No	46 (97.9%)	20 (100.0%)	66 (98.5%)	
Previous tetanus vaccine				0.116
- No	29 (61.7%)	8 (40.0%)	37 (55.2%)	
- Yes	18 (38.3%)	12 (60.0%)	30 (44.8%)	
COVID Risk score				0.003
- N	46	20	66	
- Median (Range)	2.0 (0.0, 9.0)	1.0 (0.0, 3.0)	1.0 (0.0, 9.0)	
- Mean (SD)	2.2 (1.9)	1.0 (0.9)	1.8 (1.7)	
Vaccine type				0.666
- Pfizer	34 (72.3%)	17 (85.0%)	51 (76.1%)	
- Moderna	12 (25.5%)	3 (15.0%)	15 (22.4%)	
- Johnson&Johnson	1 (2.1%)	0 (0.0%)	1 (1.5%)	
ECOG				0.004
- 0	31 (66.0%)	20 (100.0%)	51 (76.1%)	
- 1	15 (31.9%)	0 (0.0%)	15 (22.4%)	
- 2	1 (2.1%)	0 (0.0%)	1 (1.5%)	
Cancer Type				
- Breast cancer	31 (66.0%)	0	31 (66.0%)	
- GI cancer	11 (23.4%)	0	11 (23.4%)	
- Lung cancer	1 (2.1%)	0	1 (2.1%)	
- Other	4 (8.5%)	0	4 (8.5%)	
Cancer Stage				
- 1	2 (4.3%)	0	2 (4.3%)	
- 2	11 (23.4%)	0	11 (23.4%)	
- 3	10 (21.3%)	0	10 (21.3%)	
- 4	24 (51.1%)	0	24 (51.1%)	
Cancer stage				
- Stages I-III	23 (48.9%)	0	23 (48.9%)	
- Stage IV	24 (51.1%)	0	24 (51.1%)	
Type of chemotherapy				
- Multiple agent	30 (63.8%)	0	30 (63.8%)	
- Single agent	17 (36.2%)	0	17 (36.2%)	

### Humoral responses to SARS-CoV-2 vaccines

**Figure 1:**
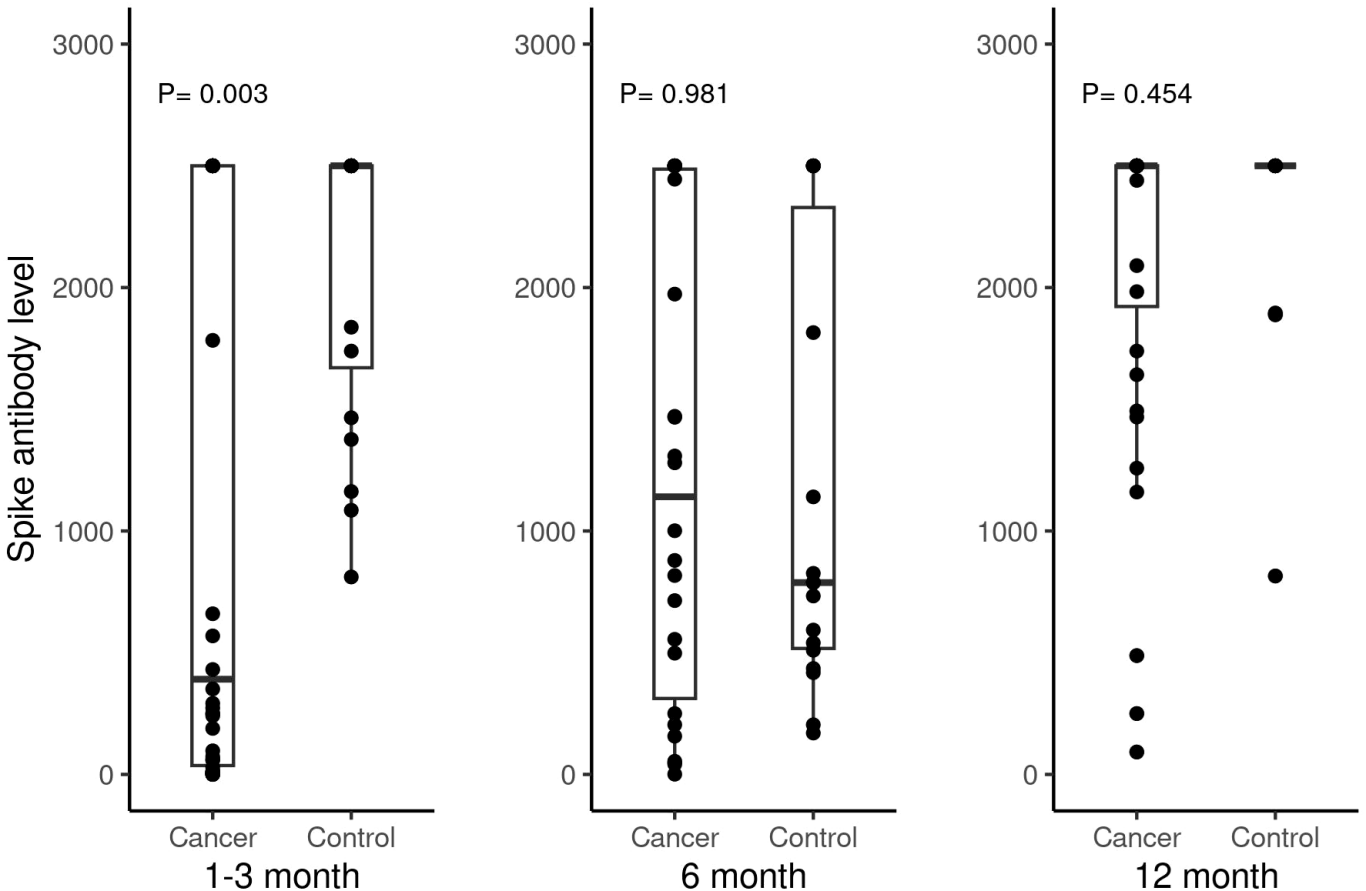
Spike antibody levels (units/ml) in cancer patients versus non-cancer healthy controls at 1-3 months, 6 months and 12 months after second doses of SARS-COVID-2 vaccinations.

**Figure 2:**
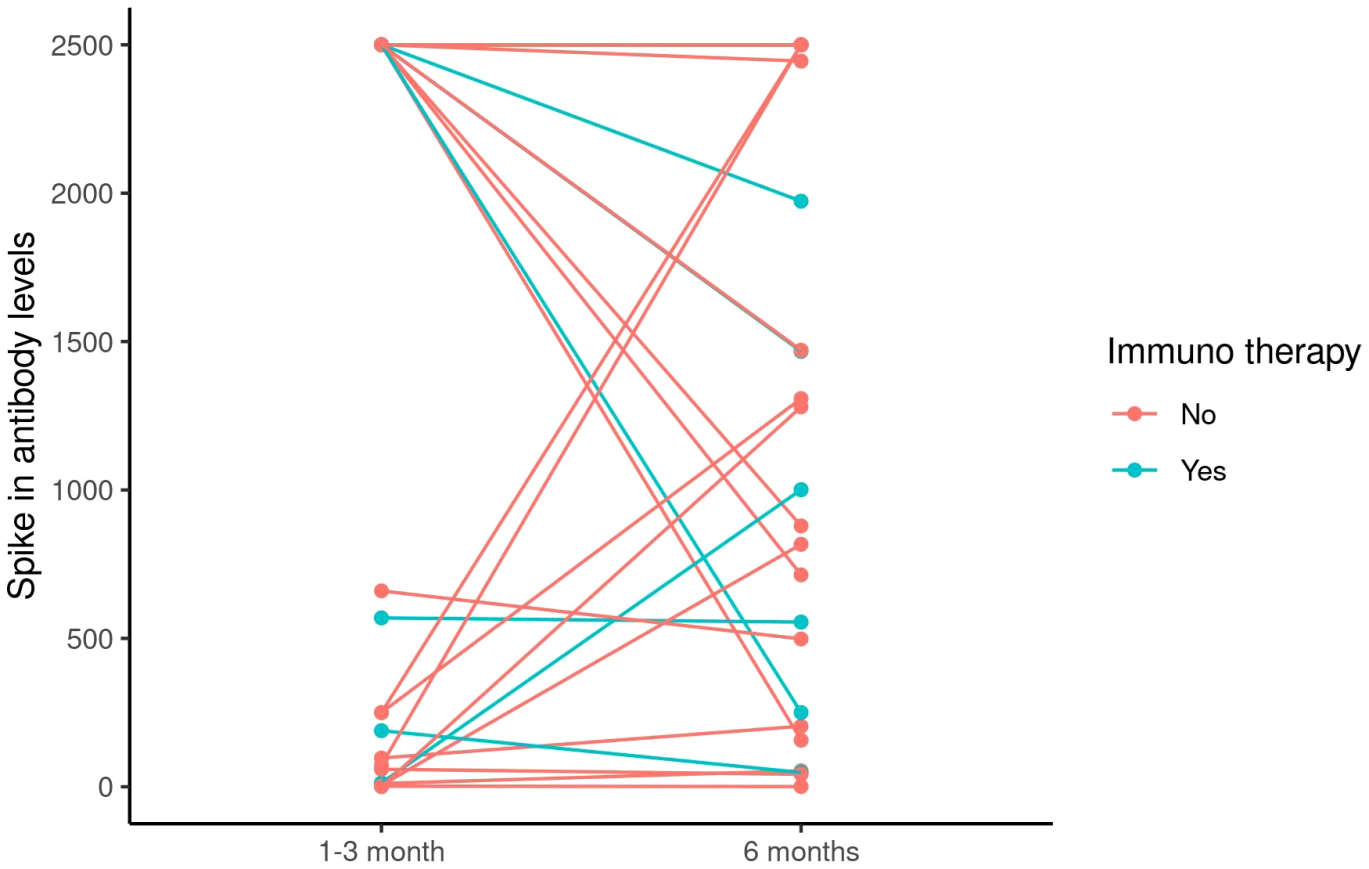
The change in spike antibody levels between 1-3 months and 6 months after second doses of SARS-COVID-2 vaccinations in cancer patients who received chemotherapy in combination with immunotherapy versus cancer patients who received chemotherapy alone.

We first evaluated the humoral immune response to the SARS-COVID-2 vaccines by measuring the SARS-CoV-2 spike antibody at baseline before the first vaccination, before the second vaccination, 1–3 months, 6 months, and 12 months after the second vaccination. The majority of patients did not have blood draws for the baseline and second timepoint prior to the second vaccination. At the 1–3 month time point, 44 out of 46 (96%) evaluable patients with cancer and 20 (100%) healthy individuals demonstrated a positive humoral response with SARS-CoV-2 spike antibody level > 0.8 units/ml per Mayo Clinic laboratory reference range (*P* = 1.00). While the positivity rate was not significantly different when comparing SARS-CoV-2 spike antibodies at the 1–3 month time point, patients with cancer had significantly lower SARS-CoV-2 spike antibodies compared to healthy individuals (*P* = 0.003, **Table 2A**, **Figure 1**). However, this significant difference did not persist at 6 months (*P* = 0.981, **Table 3A**, **Figure 1**). As shown in **Table 3A**, the median spike antibody level was 1140.5 ug/ml in patients with cancer compared to 788 ug/ml in heathy control cohort (*P* = 0.981). After excluding patients who received booster vaccination, there was also no significant difference at 12 months’ time points (*P* = 0.454, **Table 4A**, **Figure 1**). Comparisons between patients’ vaccine type, age, cancer stage, and chemotherapy type did not yield significant differences in spike antibody levels (**Tables 2B**, **3B**, **3C**, **3D**, **3E**, **4B**, **4C**, **4D**, **4E**) at any time post-vaccination. The absolute lowest lymphocyte count (ALC) at baseline was found to have a near-significant association with increased spike antibody levels at 1–3 months post-vaccination (*P* = 0.054). No association was observed between the absolute lowest neutrophil count (ANC) (*P* = 0.715), ANC/ALC ratio (*P* = 0.694) and spike antibody level. Checkpoint inhibitor immunotherapy is known to enhance antitumor immunity in some patients. However, whether combining checkpoint inhibitors with chemotherapy yields a stronger vaccine-induced humoral response remains unclear. To explore this question, we investigated antibody responses between patients who received chemotherapy alone and those who received chemotherapy in combination with checkpoint inhibitor immunotherapy. As shown in **Figure 2**, we did not observe differences between the two groups, likely due in part to the limited sample size. Larger studies are warranted to address this question.

**Table 2A:** 1–3 month data by cohort.

Immune responses	Patients with cancer (N = 47)	Healthy control (N = 20)	Total (N = 67)	p value
SARS-CoV2 Spike Antibody 1 month after last vaccine				0.003
- N	46	20	66	
- Median (Range)	391.0 (0.0, 2500.0)	2500.0 (811.0, 2500.0)	1810.0 (0.0, 2500.0)	
- Mean (SD)	1155.7 (1173.4)	2098.8 (597.3)	1441.5 (1117.3)	
Spike T cells response 1 month after last vaccine				0.167
- N	44	20	64	
- Median (Range)	224.6 (0.0, 4730.7)	379.5 (0.0, 2907.0)	292.0 (0.0, 4730.7)	
- Mean (SD)	528.3 (840.8)	682.8 (780.0)	576.6 (819.3)	
Positive humoral response				1.000
- Non-positive	2 (4.3%)	0 (0.0%)	2 (3.0%)	
- positive	44 (95.7%)	20 (100.0%)	64 (97.0%)	

**Table 2B:** The association between antibody levels at 1–3 month and baseline cancer variables for cancer patients.

Baseline characteristics	Spike antibody level ≤ 500 units/ml (N = 24)	Spike antibody level > 500 units/ml (N = 22)	Total (N = 46)	p value
Vaccine type (excluding J&J)				1.000
- Pfizer	18 (75.0%)	15 (71.4%)	33 (73.3%)	
- Moderna	6 (25.0%)	6 (28.6%)	12 (26.7%)	
Age at 1st vaccine (>55)				0.768
-<=55	11 (45.8%)	12 (54.5%)	23 (50.0%)	
- >55	13 (54.2%)	10 (45.5%)	23 (50.0%)	
Cancer stage				0.768
- Stages I-III	13 (54.2%)	10 (45.5%)	23 (50.0%)	
- Stage IV	11 (45.8%)	12 (54.5%)	23 (50.0%)	
Type of chemotherapy				0.538
- Multiple agent	17 (70.8%)	13 (59.1%)	30 (65.2%)	
- Single agent	7 (29.2%)	9 (40.9%)	16 (34.8%)	
Lowest absolute neutrophil count in between the vaccine				0.715
- N	20	20	40	
- Median (Range)	2.2 (0.1, 8.4)	2.6 (0.8, 8.2)	2.3 (0.1, 8.4)	
- Mean (SD)	3.0 (2.2)	3.1 (2.0)	3.0 (2.1)	
Lowest absolute lymphocyte count in between the vaccine				0.054
- N	8	7	15	
- Median (Range)	1.1 (0.4, 1.9)	1.6 (0.8, 2.5)	1.5 (0.4, 2.5)	
- Mean (SD)	1.2 (0.5)	1.7 (0.6)	1.4 (0.6)	
ANC/ALC				0.694
- N	8	7	15	
- Median (Range)	2.5 (0.1, 6.4)	1.8 (0.7, 2.4)	1.8 (0.1, 6.4)	
- Mean (SD)	2.8 (2.4)	1.6 (0.6)	2.2 (1.9)	

**Table 3A:** 6-month data by cohort.

Immune responses	Patients with cancer (N = 34)	Healthy control (N = 20)	Total (N = 54)	p value
Post vaccine Spike antibody concentrations (ug/ml)				0.981
- N	26	18	44	
- Median (Range)	1140.5 (0.8, 2500.0)	788.0 (170.0, 2500.0)	852.5 (0.8, 2500.0)	
- Mean (SD)	1256.3 (977.6)	1192.2 (909.4)	1230.1 (940.0)	
Post vaccine Spike T cell levels (per million PBMC)				0.207
- N	19	18	37	
- Median (Range)	360.0 (1.4, 1498.7)	137.5 (0.0, 1458.0)	180.0 (0.0, 1498.7)	
- Mean (SD)	446.4 (463.8)	272.8 (387.6)	362.0 (431.6)	
Change of antibody from 1m to 6m				0.015
- N	26	18	44	
- Median (Range)	-0.6 (-2343.0, 2427.0)	-821.0 (-1960.0, 0.0)	-98.0 (-2343.0, 2427.0)	
- Mean (SD)	-77.5 (1166.6)	-904.2 (750.9)	-415.7 (1087.8)	
Change of T cell from 1m to 6m				0.988
- N	19	18	37	
- Median (Range)	-234.0 (-3296.0, 568.0)	-106.5 (-2292.0, 315.0)	-125.0 (-3296.0, 568.0)	
- Mean (SD)	-403.8 (827.8)	-430.4 (735.3)	-416.7 (773.4)	

*Change of antibody from 1m to 6m: p=0.59 for cancer patients, p=0.002 for control patients.

*Change of T cell from 1m to 6m: p=0.02 for cancer patients;p=0.02 for control patients.

*This analysis excluded patients with 3 or more vaccinations before 6 months.

**Table 3B:** 6-month data by vaccine type.

Immune responses	Pfizer (N = 43)	Moderna (N = 10)	Total (N = 53)	p value
Post vaccine Spike antibody concentrations (ug/ml)				0.383
- N	34	9	43	
- Median (Range)	788.0 (0.8, 2500.0)	1308.0 (48.0, 2500.0)	826.0 (0.8, 2500.0)	
- Mean (SD)	1136.2 (936.2)	1443.8 (918.4)	1200.6 (930.3)	
Post vaccine Spike T cell levels (per million PBMC)				0.099
- N	30	7	37	
- Median (Range)	291.3 (0.0, 1498.7)	113.3 (1.4, 360.0)	180.0 (0.0, 1498.7)	
- Mean (SD)	419.4 (458.3)	115.8 (121.9)	362.0 (431.6)	
Change of antibody from 1m to 6m				0.280
- N	34	9	43	
- Median (Range)	-414.0 (-2343.0, 2427.0)	-55.0 (-1621.0, 1058.0)	-141.0 (-2343.0, 2427.0)	
- Mean (SD)	-519.8 (1155.4)	-68.7 (806.2)	-425.4 (1098.7)	
Change of T cell from 1m to 6m				0.128
- N	30	7	37	
- Median (Range)	-94.8 (-3296.0, 568.0)	-290.7 (-2292.0, 0.5)	-125.0 (-3296.0, 568.0)	
- Mean (SD)	-364.3 (776.6)	-641.5 (775.3)	-416.7 (773.4)	

*This analysis excluded patients with 3 or more vaccinations before 6 months.

**Table 3C:** 6-month data by age.

Immune responses	Age ≤ 55 (N = 27)	Age > 55 (N = 27)	Total (N = 54)	p value
Post vaccine Spike antibody concentrations (ug/ml)				0.104
- N	25	19	44	
- Median (Range)	1280.0 (48.0, 2500.0)	593.0 (0.8, 2500.0)	852.5 (0.8, 2500.0)	
- Mean (SD)	1375.6 (879.7)	1038.6 (1005.5)	1230.1 (940.0)	
Post vaccine Spike T cell levels (per million PBMC)				0.189
- N	15	22	37	
- Median (Range)	123.0 (0.0, 1458.0)	360.0 (0.0, 1498.7)	180.0 (0.0, 1498.7)	
- Mean (SD)	239.8 (366.4)	445.3 (460.3)	362.0 (431.6)	
Change of antibody from 1m to 6m				0.289
- N	25	19	44	
- Median (Range)	-141.0 (-1960.0, 2427.0)	-55.0 (-2343.0, 988.0)	-98.0 (-2343.0, 2427.0)	
- Mean (SD)	-231.5 (1165.9)	-658.1 (951.3)	-415.7 (1087.8)	
Change of T cell from 1m to 6m				0.703
- N	15	22	37	
- Median (Range)	-133.0 (-778.0, 315.0)	-103.6 (-3296.0, 568.0)	-125.0 (-3296.0, 568.0)	
- Mean (SD)	-202.4 (334.1)	-562.8 (946.4)	-416.7 (773.4)	

*This analysis excluded patients with 3 or more vaccinations before 6 months.

**Table 3D:** 6-month data by cancer stage.

Immune responses	Stages I-III (N = 15)	Stage IV (N = 19)	Total (N = 34)	p value
Post vaccine Spike antibody concentrations (ug/ml)				0.500
- N	12	14	26	
- Median (Range)	1079.5 (0.8, 2500.0)	1236.0 (42.0, 2500.0)	1140.5 (0.8, 2500.0)	
- Mean (SD)	1145.9 (944.7)	1350.9 (1030.5)	1256.3 (977.6)	
Post vaccine Spike T cell levels (per million PBMC)				0.310
- N	8	11	19	
- Median (Range)	291.3 (3.6, 930.7)	434.7 (1.4, 1498.7)	360.0 (1.4, 1498.7)	
- Mean (SD)	312.8 (319.0)	543.6 (539.7)	446.4 (463.8)	
Change of antibody from 1m to 6m				0.918
- N	12	14	26	
- Median (Range)	-7.6 (-1786.0, 2427.0)	0.0 (-2343.0, 2250.0)	-0.6 (-2343.0, 2427.0)	
- Mean (SD)	13.0 (1189.1)	-155.1 (1186.1)	-77.5 (1166.6)	
Change of T cell from 1m to 6m				0.492
- N	8	11	19	
- Median (Range)	-264.7 (-1129.3, 222.7)	-101.6 (-3296.0, 568.0)	-234.0 (-3296.0, 568.0)	
- Mean (SD)	-340.3 (412.9)	-450.0 (1052.9)	-403.8 (827.8)	

*This analysis excluded patients with 3 or more vaccinations before 6 months.

**Table 3E:** 6-month data by chemotherapy type.

Immune responses	Multiple agent (N = 22)	Single agent (N = 12)	Total (N = 34)	p value
Post vaccine Spike antibody concentrations (ug/ml)				0.479
- N	15	11	26	
- Median (Range)	1308.0 (48.0, 2500.0)	817.0 (0.8, 2500.0)	1140.5 (0.8, 2500.0)	
- Mean (SD)	1329.5 (912.0)	1156.5 (1098.2)	1256.3 (977.6)	
Post vaccine Spike T cell levels (per million PBMC)				0.717
- N	11	8	19	
- Median (Range)	390.7 (3.6, 1498.7)	338.0 (1.4, 981.3)	360.0 (1.4, 1498.7)	
- Mean (SD)	487.1 (528.6)	390.5 (384.5)	446.4 (463.8)	
Change of antibody from 1m to 6m				0.310
- N	15	11	26	
- Median (Range)	-141.0 (-2250.0, 2250.0)	0.0 (-2343.0, 2427.0)	-0.6 (-2343.0, 2427.0)	
- Mean (SD)	-261.2 (1199.0)	173.1 (1127.0)	-77.5 (1166.6)	
Change of T cell from 1m to 6m				0.177
- N	11	8	19	
- Median (Range)	-290.7 (-3296.0, 376.0)	-50.6 (-741.3, 568.0)	-234.0 (-3296.0, 568.0)	
- Mean (SD)	-617.7 (993.2)	-109.7 (426.9)	-403.8 (827.8)	

* This analysis excluded patients with 3 or more vaccinations before 6 months.

**Table 4A:** 12-month data by cohort.

Immune responses	Patients with cancer (N = 47)	Healthy control (N = 20)	Total (N = 67)	p value
SARS-CoV2 Spike Antibody 12 months after last vaccine				0.454
- N	36	13	49	
- Median (Range)	2500.0 (92.0, 2500.0)	2500.0 (815.0, 2500.0)	2500.0 (92.0, 2500.0)	
- Mean (SD)	2114.0 (691.1)	2276.8 (494.3)	2157.2 (643.9)	
Spike.T.Cell.12m				0.077
- N	28	12	40	
- Median (Range)	112.7 (0.0, 1477.3)	209.5 (37.3, 1314.7)	157.3 (0.0, 1477.3)	
- Mean (SD)	232.8 (343.9)	345.5 (357.9)	266.6 (347.5)	
Change of antibody from 1m to 12m				0.125
- N	36	13	49	
- Median (Range)	719.5 (-2250.0, 2500.0)	0.0 (-650.0, 1689.0)	0.0 (-2250.0, 2500.0)	
- Mean (SD)	919.6 (1342.1)	284.5 (640.8)	751.1 (1223.2)	
Change of T cell from 1m to 12m				0.243
- N	26	12	38	
- Median (Range)	-1.3 (-4358.0, 1366.7)	-143.3 (-2057.3, 640.0)	-63.3 (-4358.0, 1366.7)	
- Mean (SD)	-253.2 (983.3)	-372.5 (784.9)	-290.9 (916.3)	
Change of antibody from 6m to 12m				0.407
- N	36	13	49	
- Median (Range)	13.5 (-1007.0, 2499.2)	1713.0 (0.0, 2065.0)	362.0 (-1007.0, 2499.2)	
- Mean (SD)	775.8 (1149.8)	1122.0 (896.6)	867.6 (1090.3)	
Change of T cell from 6m to 12m				0.131
- N	14	12	26	
- Median (Range)	-15.9 (-1062.0, 303.1)	67.8 (-1144.2, 541.0)	2.9 (-1144.2, 541.0)	
- Mean (SD)	-155.3 (396.4)	19.1 (415.9)	-74.8 (407.0)	
% Neutralization				0.423
- N	34	12	46	
- Median (Range)	45.9 (0.0, 100.0)	56.6 (0.0, 85.4)	51.0 (0.0, 100.0)	
- Mean (SD)	49.9 (28.9)	39.4 (35.1)	47.2 (30.6)	

*Change of antibody: p<0.001 from 1m to 12m, p=0.001 from 6m to 12m for cancer patients; p=0.178 from 1m to 12m, p= 0.009 from 6m to 12m for controls.

*Change of T cell: p=0.408 from 1m to 12m, p=0.296 from 6m to 12m for cancer patients; p=0.092 from 1m to 12m, p=0.301 from 6m to 12m for controls.

**Table 4B:** 12-month data by vaccine type.

Immune responses	Pfizer (N = 51)	Moderna (N = 15)	Total (N = 66)	p value
SARS-CoV2 Spike Antibody 12 months after last vaccine				0.602
- N	36	12	48	
- Median (Range)	2500.0 (250.0, 2500.0)	2500.0 (92.0, 2500.0)	2500.0 (92.0, 2500.0)	
- Mean (SD)	2211.8 (553.0)	1964.8 (880.6)	2150.0 (648.7)	
Spike.T.Cell.12m				0.826
- N	32	7	39	
- Median (Range)	164.7 (0.0, 1477.3)	153.3 (20.0, 1134.0)	161.3 (0.0, 1477.3)	
- Mean (SD)	267.6 (346.0)	293.0 (396.9)	272.2 (350.2)	
Change of antibody from 1m to 12m				0.752
- N	36	12	48	
- Median (Range)	0.0 (-2250.0, 2500.0)	160.1 (0.0, 2311.0)	41.1 (-2250.0, 2500.0)	
- Mean (SD)	753.4 (1317.0)	806.9 (977.5)	766.8 (1231.2)	
Change of T cell from 1m to 12m				0.266
- N	32	5	37	
- Median (Range)	-90.0 (-4358.0, 1366.7)	31.1 (-2057.3, 303.3)	-78.7 (-4358.0, 1366.7)	
- Mean (SD)	-300.2 (933.6)	-293.1 (993.8)	-299.2 (927.5)	
Change of antibody from 6m to 12m				0.116
- N	36	12	48	
- Median (Range)	1169.0 (-1007.0, 2499.2)	0.0 (-820.0, 2452.0)	379.5 (-1007.0, 2499.2)	
- Mean (SD)	1048.2 (1074.7)	398.1 (1047.3)	885.7 (1094.4)	
Change of T cell from 6m to 12m				0.095
- N	23	3	26	
- Median (Range)	-7.7 (-1144.2, 541.0)	234.7 (30.6, 303.1)	2.9 (-1144.2, 541.0)	
- Mean (SD)	-109.3 (419.1)	189.5 (141.8)	-74.8 (407.0)	
% Neutralization				0.722
- N	35	10	45	
- Median (Range)	58.0 (0.0, 100.0)	41.9 (0.0, 89.6)	49.0 (0.0, 100.0)	
- Mean (SD)	47.1 (32.6)	45.2 (24.6)	46.7 (30.8)	

**Table 4C:** 12-month data by age.

Immune responses	Age ≤ 55 (N = 35)	Age > 55 (N = 32)	Total (N = 67)	p value
SARS-CoV2 Spike Antibody 12 months after last vaccine				0.862
- N	28	21	49	
- Median (Range)	2500.0 (92.0, 2500.0)	2500.0 (1258.0, 2500.0)	2500.0 (92.0, 2500.0)	
- Mean (SD)	2064.6 (779.5)	2280.5 (383.3)	2157.2 (643.9)	
Spike.T.Cell.12m				0.914
- N	21	19	40	
- Median (Range)	161.3 (0.0, 1477.3)	153.3 (6.9, 1314.7)	157.3 (0.0, 1477.3)	
- Mean (SD)	295.7 (389.3)	234.4 (302.0)	266.6 (347.5)	
Change of antibody from 1m to 12m				0.462
- N	28	21	49	
- Median (Range)	0.0 (-2250.0, 2494.8)	803.0 (-517.0, 2500.0)	0.0 (-2250.0, 2500.0)	
- Mean (SD)	611.1 (1298.3)	937.9 (1118.7)	751.1 (1223.2)	
Change of T cell from 1m to 12m				0.271
- N	19	19	38	
- Median (Range)	-34.7 (-829.2, 1366.7)	-83.6 (-4358.0, 266.7)	-63.3 (-4358.0, 1366.7)	
- Mean (SD)	13.8 (473.2)	-595.6 (1142.8)	-290.9 (916.3)	
Change of antibody from 6m to 12m				0.123
- N	28	21	49	
- Median (Range)	0.5 (-820.0, 2452.0)	1718.0 (-1007.0, 2499.2)	362.0 (-1007.0, 2499.2)	
- Mean (SD)	650.3 (961.7)	1157.4 (1204.3)	867.6 (1090.3)	
Change of T cell from 6m to 12m				0.421
- N	10	16	26	
- Median (Range)	47.7 (-1144.2, 541.0)	-3.4 (-1062.0, 303.1)	2.9 (-1144.2, 541.0)	
- Mean (SD)	6.5 (443.3)	-125.6 (388.6)	-74.8 (407.0)	
% Neutralization				0.527
- N	26	20	46	
- Median (Range)	59.2 (0.0, 100.0)	45.9 (0.0, 100.0)	51.0 (0.0, 100.0)	
- Mean (SD)	48.9 (32.6)	44.9 (28.5)	47.2 (30.6)	

**Table 4D:** 12-month data by cancer stage.

Immune responses	Stages I-III (N = 23)	Stage IV (N = 24)	Total (N = 47)	p value
SARS-CoV2 Spike Antibody 12 months after last vaccine				0.610
- N	19	17	36	
- Median (Range)	2500.0 (92.0, 2500.0)	2500.0 (250.0, 2500.0)	2500.0 (92.0, 2500.0)	
- Mean (SD)	2047.6 (731.1)	2188.1 (657.6)	2114.0 (691.1)	
Spike.T.Cell.12m				0.550
- N	14	14	28	
- Median (Range)	128.7 (9.8, 1134.0)	85.4 (0.0, 1477.3)	112.7 (0.0, 1477.3)	
- Mean (SD)	240.6 (315.1)	225.0 (382.4)	232.8 (343.9)	
Change of antibody from 1m to 12m				0.154
- N	19	17	36	
- Median (Range)	1641.6 (-1032.0, 2500.0)	0.0 (-2250.0, 2494.8)	719.5 (-2250.0, 2500.0)	
- Mean (SD)	1171.9 (1234.9)	637.7 (1436.8)	919.6 (1342.1)	
Change of T cell from 1m to 12m				0.479
- N	13	13	26	
- Median (Range)	-83.6 (-984.7, 303.3)	17.2 (-4358.0, 1366.7)	-1.3 (-4358.0, 1366.7)	
- Mean (SD)	-148.6 (335.1)	-357.9 (1370.5)	-253.2 (983.3)	
Change of antibody from 6m to 12m				0.747
- N	19	17	36	
- Median (Range)	26.0 (-1007.0, 2499.2)	0.0 (-311.0, 2458.0)	13.5 (-1007.0, 2499.2)	
- Mean (SD)	783.8 (1264.7)	766.8 (1045.2)	775.8 (1149.8)	
Change of T cell from 6m to 12m				0.710
- N	7	7	14	
- Median (Range)	-16.0 (-870.7, 215.1)	-15.7 (-1062.0, 303.1)	-15.9 (-1062.0, 303.1)	
- Mean (SD)	-185.5 (363.8)	-125.1 (453.9)	-155.3 (396.4)	
% Neutralization				0.445
- N	19	15	34	
- Median (Range)	40.9 (0.0, 100.0)	58.0 (13.2, 100.0)	45.9 (0.0, 100.0)	
- Mean (SD)	45.4 (28.9)	55.7 (28.9)	49.9 (28.9)	

**Table 4E:** 12-month data by chemotherapy type.

Immune responses	Multiple agent (N = 30)	Single agent (N = 17)	Total (N = 47)	p value
SARS-CoV2 Spike Antibody 12 months after last vaccine				0.254
- N	22	14	36	
- Median (Range)	2500.0 (92.0, 2500.0)	2500.0 (250.0, 2500.0)	2500.0 (92.0, 2500.0)	
- Mean (SD)	2029.8 (710.7)	2246.3 (662.6)	2114.0 (691.1)	
Spike.T.Cell.12m				0.908
- N	16	12	28	
- Median (Range)	137.3 (2.7, 1134.0)	78.7 (0.0, 1477.3)	112.7 (0.0, 1477.3)	
- Mean (SD)	213.2 (287.6)	258.9 (419.8)	232.8 (343.9)	
Change of antibody from 1m to 12m				0.491
- N	22	14	36	
- Median (Range)	160.1 (-1340.0, 2500.0)	1588.0 (-2250.0, 2498.0)	719.5 (-2250.0, 2500.0)	
- Mean (SD)	806.8 (1268.2)	1096.9 (1481.9)	919.6 (1342.1)	
Change of T cell from 1m to 12m				0.494
- N	14	12	26	
- Median (Range)	-19.3 (-4358.0, 303.3)	6.7 (-984.7, 1366.7)	-1.3 (-4358.0, 1366.7)	
- Mean (SD)	-459.2 (1212.0)	-12.9 (587.1)	-253.2 (983.3)	
Change of antibody from 6m to 12m				0.574
- N	22	14	36	
- Median (Range)	13.5 (-1007.0, 2452.0)	128.5 (-60.0, 2499.2)	13.5 (-1007.0, 2499.2)	
- Mean (SD)	659.4 (1167.8)	958.7 (1139.1)	775.8 (1149.8)	
Change of T cell from 6m to 12m				0.620
- N	7	7	14	
- Median (Range)	-16.0 (-1062.0, 215.1)	-15.7 (-366.7, 303.1)	-15.9 (-1062.0, 303.1)	
- Mean (SD)	-279.5 (493.3)	-31.0 (247.2)	-155.3 (396.4)	
% Neutralization				0.535
- N	21	13	34	
- Median (Range)	40.9 (0.0, 100.0)	58.3 (0.7, 98.8)	45.9 (0.0, 100.0)	
- Mean (SD)	48.5 (29.9)	52.2 (28.3)	49.9 (28.9)	

### Cellular response to SARS-CoV-2 mRNA vaccines

**Figure 3:**
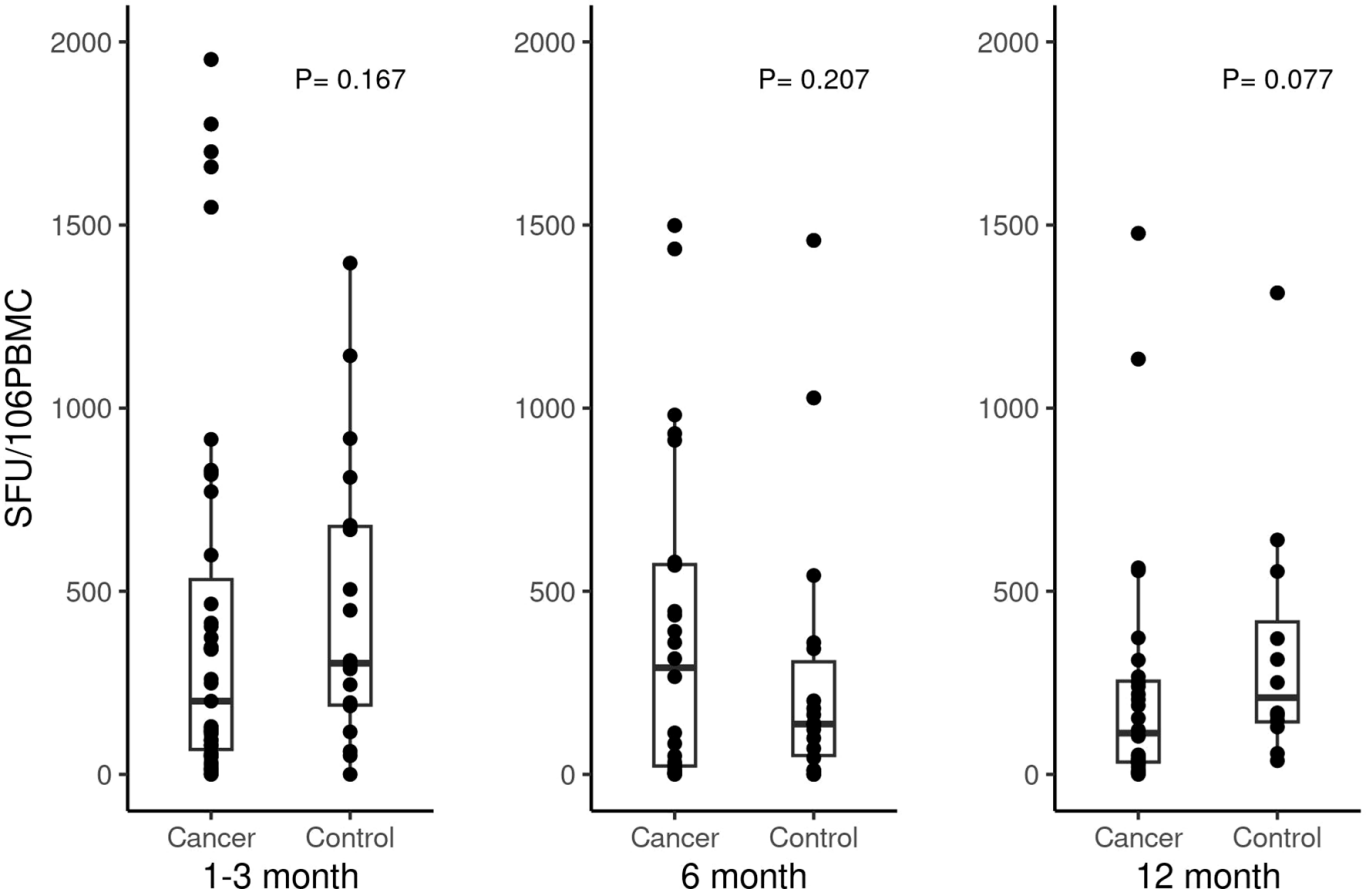
Antigen specific T cell response (SFU/106 PBMC) in cancer patients versus non-cancer healthy controls at 1-3 months, 6 months and 12 months after second doses of SARS-COVID-2 vaccinations.

Besides the humoral response, we further studied the antigen-specific T cell response to SARS-CoV-2 mRNA vaccines at 1–3 months, 6 and 12 months post vaccinations. Spike T cell counts were not found to be significantly different between patients with cancer and healthy individuals at 1–3 months (*P* = 0.167), 6-months (*P* = 0.207), and 12 months (*P* = 0.077) (**Figure 3**) after the second vaccination. Of note, three participants who had spike T cell >2000 SFU/10^6^ PBMC at 1–3 months were included in the analysis but not shown in **Figure 3** in order to keep consistent scale across different time points. It included two healthy controls who had spike T-cells of 2907 and 2428 SFU/10^6^ PBMC and one cancer patient receiving chemotherapy in combination with immunotherapy who had spike T- cells of 4731SFU/10^6^ PBMC. No participants had spike-specific T cells >2000 SFU/10^6^ PBMC at 6 months or 12 months.

### Longitudinal changes between 1–3 month and 6-month time point

**Figure 4:**
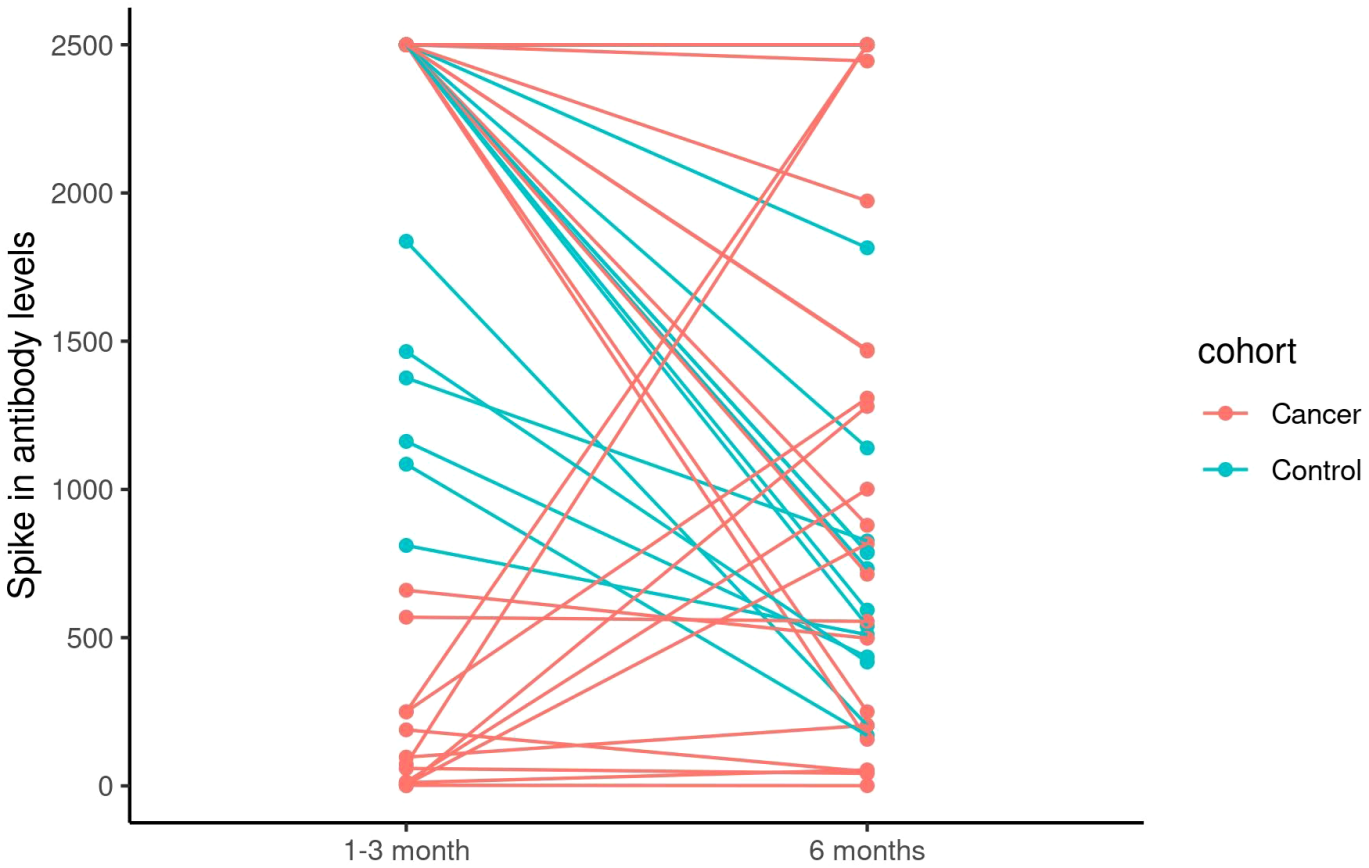
The change in spike antibody levels between 1-3 months and 6 months after second doses of SARS-COVID-2 vaccinations in cancer patients versus non-cancer healthy controls.

Given that the significant difference in the SARS-CoV-2 spike antibody at the 1–3 month time point no longer persists at the 6-month time point, we further evaluated the changes between 1–3 month and 6-month time points. For this analysis, we excluded 3 patients who received the additional booster vaccination with a total of three vaccinations prior to the 6 month time point as these patients were expected to have increased immune responses due to the additional booster vaccine. Between the 1–3 month and 6-month time points, changes in spike antibody levels for the cancer patient (median -0.6) and healthy individual (median -821.0) cohorts were found to be significantly different (*P* = 0.015). Whereas healthy individuals all experienced a decrease or no change in spike antibody levels (range -1960.0, 0), eight patients with cancer experienced an increase (range -2343.0, 2427.0) in their spike antibody levels (**Figure 4**). This difference in changing antibody levels between the cancer and healthy cohorts was further evaluated for the 6–12 month period. However, the tendency for patients with cancer to experience significantly greater increases in spike antibody levels compared to healthy individuals did not persist (*P* = 0.407, **Table 4A**). Furthermore, analysis for a 1–12 month period showed no overall difference in changing antibody levels between cancer and healthy cohorts across the full study period (*P* = 0.125, **Table 4A**). No significant correlations between spike T cell counts and any baseline characteristics were observed during the 1–6 month, 6–12 month, or 1–12 month periods.

### Neutralizing antibodies against omicron variant

**Figure 5:**
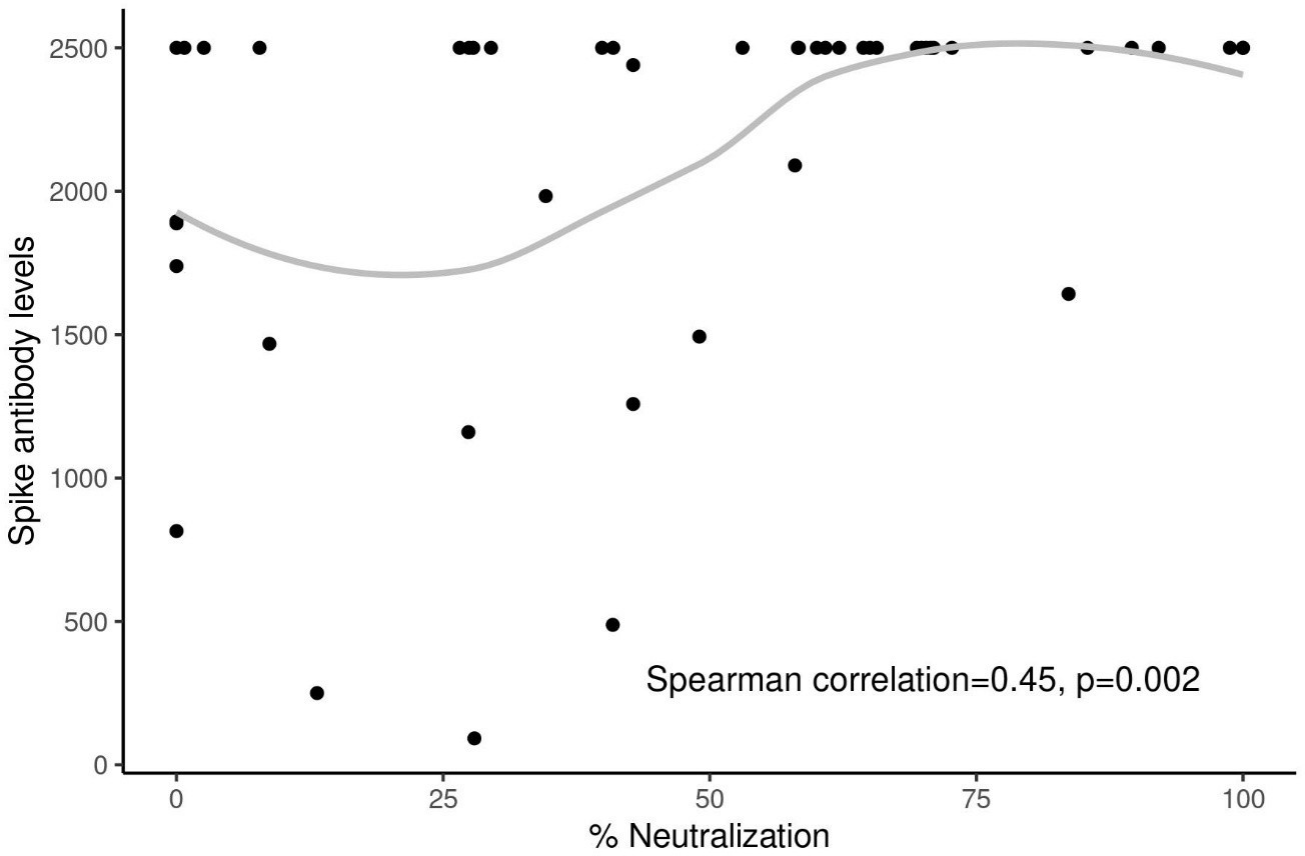
Correlation between neutralizing antibodies against Omicron variant and spike antibody levels

We further evaluated the neutralizing antibodies against the Omicron variant, which was the new prevalent variant at that time. While there were numerically more neutralizing antibody responses in healthy individuals (median 56.6%) compared to patients with cancer (median 45.9%), this difference was not statistically significant (*P* = 0.423). Similarly, there were no significant differences in neutralizing antibodies against the Omicron variant by vaccine type (*P* = 0.722), age (*P* = 0.527), cancer stage (*P* = 0.445), and chemotherapy type (*P* = 0.535). Patients with greater % neutralizing antibodies against the new variant tended to also have significantly higher spike antibody levels, with a Spearman correlation of 0.45 (*P* = 0.002, **Figure 5**).

### Patient outcomes and hospitalization

No significant grade 3 or above adverse events were observed in patients with cancer receiving SARS-CoV-2 vaccination prior to or during cytotoxic chemotherapy. Most adverse events were as expected based on previous studies (14). During the study period, there were numerically more patients in the cancer cohort who developed self-reported COVID-19 infection, with 5 patients (10.9%) in the cancer cohort compared to 2 patients (10%) in the healthy cohort. However, this difference was not statistically significant (**Table 5**, *P* = 1.000). One cancer patient (12.5%) was hospitalized compared to 0 patients in the healthy cohort (*P* = 1.000). All vaccinated patients with COVID-19 infection in our cohort recovered from the infection.

**Table 5:** COVID infection at 12 months.

COVID-19 status	Patients with cancerN = 47)	Healthy control (N = 20)	Total (N = 67)	p value
COVID-19 infection				1.000
- No	41 (89.1%)	18 (90.0%)	59 (89.4%)	
- Yes	5 (10.9%)	2 (10.0%)	7 (10.6%)	
Hospitalization				1.000
- No	4 (80.0%)	2 (100.0%)	6 (85.7%)	
- Yes	1 (20.0%)	0 (0.0%)	1 (14.3%)	
Time from 1st vac to infection (months)				0.857
- N	5	2	7	
- Median (Range)	9.0 (6.7, 11.3)	9.8 (7.3, 12.3)	9.0 (6.7, 12.3)	
- Mean (SD)	9.3 (1.8)	9.8 (3.6)	9.4 (2.1)	

* % hospitalization was calculated based on patients with infection only.

## Discussion

Although SARS-CoV-2 vaccines have been widely adopted in the community, leading to a significant reduction of COVID-19 infection, related hospitalization, and death, their long-term effectiveness in patients with cancer remains less clear. Patients with cancer are known to be more vulnerable to infections, including COVID-19, due to immune suppressive conditions from cancer itself, treatments, and comorbidities (32). Although SARS-CoV-2 vaccines are found to be highly effective in healthy individuals, the extent and long-term durability of antibody and T-cell-specific cellular responses in patients with solid cancers receiving chemotherapy remain less known.

Several studies have reported lower seroconversion rates in patients with cancer receiving treatment, particularly among those with hematologic malignancies and those receiving anti-CD20 monoclonal antibody therapy (22–24). Thakkar et al. (33) examined anti-spike IgG titers in 242 patients with cancer, reporting an overall seroconversion rate of 94%, with 98% in solid tumor patients compared to 85% in those with hematologic malignancies. Notably, seroconversion rates were even lower in patients receiving anti-CD20 therapy (70%) and those with prior stem cell transplants (73%). However, this study had a short follow-up period of 19–53 days post-vaccination. Similarly, Linardou et al. (30) found a reduced seroconversion rate in patients with cancer compared to healthy individuals (90.5% vs. 98%). Among patients with cancer, higher antibody titers were observed in women, never-smokers, and individuals under 50 years old.

Our study evaluated both antibody and antigen-specific T-cell responses in patients with solid cancers undergoing chemotherapy compared to healthy individuals over a one-year period following vaccination. We found that while patients with cancer exhibited significantly lower spike antibody levels at 1–3 months post-vaccination compared to healthy individuals, this difference was no longer observed at the 6-month and 12-month time points. Notably, in contrast to the decline in spike antibody levels seen in healthy individuals at 6 months, patients with cancer exhibited significantly higher spike antibody levels at this time point, suggesting a delayed yet robust humoral response. Markedly, 48.9% of our patient cohort had stage I–III disease and had likely completed chemotherapy, which may have contributed to higher spike antibody levels at later time points. Of note, we excluded patients who received booster vaccinations between the 1–3-month and 6-month assessments, making it unlikely that booster doses accounted for the delayed humoral response. Additionally, no participants in either the cancer or healthy cohorts experienced a COVID-19 infection during this interval (**Table 5**). Our findings are consistent with a previous study done by Nguyen Liem et al, who similarly observed reduced humoral responses at 1 month post-vaccination and noticed increased humoral responses at later time points in patients with solid tumors compared with healthy controls (34). Our findings suggest that patients with solid malignancies may mount a delayed antibody response, particularly following the completion of therapy.

While humoral responses to SARS-CoV-2 vaccination have been extensively studied, data on T-cell responses in patients with cancer remain limited. Ehmsen et al. (35) conducted a large study involving 524 patients with cancer, assessing not only anti-spike IgG responses but also CD4/CD8 T-cell responses. The majority of participants (62%) had hematologic malignancies. Similar to previous studies, this study reported a high overall seroconversion rate of 93% and patients with hematologic malignancies had significantly lower seroconversion rates (66%). Regarding SARS-CoV-2-specific T-cell reactivity, 46% of patients with solid tumors mounted a T-cell response, with 76% generating both CD4 and CD8 T-cell responses, while 23% exhibited CD8 T-cell responses alone. Similarly, 45% of patients with hematologic malignancies developed a T-cell response, with 81% displaying both CD4 and CD8 T-cell responses and 18% exhibiting only CD8 T-cell responses. These findings suggest that patients with solid malignancies generally achieve robust humoral responses but have suboptimal cellular immune responses, whereas patients with hematologic malignancies exhibit deficiencies in both humoral and cellular immunity. However, the study had a relatively short follow-up, with a median of 36 days after the second vaccination and additional data up to three months post-vaccination. In contrast, our findings demonstrate that patients with solid malignancies are capable of eliciting antigen-specific T-cell responses comparable to those of healthy individuals, particularly at the 6- and 12-month time points, highlighting the durability of their cellular immune response over time.

Previous studies have shown that advanced age is a risk factor for a reduced antibody response following vaccination (20, 29, 30, 36). Although patients in the cancer cohort were older than those in the healthy control group, there were no significant differences in age distribution when stratified into groups older than 55 and 55 or younger. Using this age cutoff, no significant correlation was observed between age and humoral response at any time point throughout the study. Additionally, no differences in antibody levels were found among patients with cancer based on vaccine type (BNT162b2 mRNA vs. mRNA-1273), disease stage (early-stage vs. metastatic cancer), or treatment regimen (multiple-agent vs. single-agent chemotherapy). However, there was an imbalance in the cancer cohort, with a higher proportion of female patients with breast cancer. Despite the cancer cohort having a higher COVID-19 risk score, there was no significant difference in hospitalization rates between patients with cancer and healthy individuals.

In conclusion, our study demonstrated that despite initial impairments in humoral responses, patients with solid malignancies receiving chemotherapy effectively mounted long-term humoral and cellular immunity following SARS-CoV-2 vaccination. By 6 and 12 months post-vaccination, patients with cancer exhibited comparable antibody levels, T-cell responses, and neutralization capacity to healthy individuals, contributing to similar rates of infection and hospitalization. These findings suggest that while the immune response in patients with cancer may be delayed, it remains robust over time. However, the study is limited due to the overrepresentation of breast patients with cancer, female gender, and white race in the cancer cohort, as well as the small sample size, which restricted analysis of individual chemotherapy regimens. Notably, while our study assessed antigen-specific cellular immunity by quantifying spike-specific T cells per million PBMCs across multiple time points and study groups, this approach does not fully capture the functional and phenotypic heterogeneity of vaccine-induced T-cell responses. Moreover, due to limited samples, our study did not assess the establishment of immunological memory, a central component of vaccine-mediated protection. Several prior studies have investigated memory T and B lymphocytes responses to COVID-19 vaccination in patients with cancers. Favalli et al. longitudinally assessed both humoral and cellular immunity in adults with solid tumors and found a less efficient memory B-cell development in patients receiving chemotherapy compared with healthy controls. Although spike-specific memory CD4+ and CD8+ T cells were found in patients with cancers, their frequencies were reduced relative to controls, suggesting impaired activation and differentiation to immunological memory in cancer patients (37). Similarly, Shroff et al. demonstrated that spike-specific plasma cell-biased memory B cell response in patients with solid cancers after the second vaccinations were comparable to those observed in healthy controls after the first vaccination (38). Marutescu et al. reported that patients with head and neck cancers developed durable plasma SARS-CoV-2 antibody responses and circulating Tet^++^ B cells at levels comparable to controls, with sustained persistence over time (39). In addition, Yulie et al. observed comparable memory B- and T-cell responses between glioma patients and healthy volunteers (40). The heterogeneity observed in immune memory responses to COVID-19 vaccination among patients with cancer across studies likely reflects differences in cancer types, treatment exposures, vaccination timing relative to anticancer treatment, and the immunological platforms and assays used for response assessment. Future studies incorporating the memory phenotyping and functional immune assays will be critical to fully characterize long-term vaccine-induced immunity in patients with cancers. Larger studies with more diverse patient groups are needed to validate these findings and further investigate the impact of different chemotherapy types on vaccine-induced immunity.

## Data Availability

The raw data supporting the conclusions of this article will be made available by the authors, without undue reservation.
